# Targeted surveillance reveals native and invasive mosquito species infected with Usutu virus

**DOI:** 10.1186/s13071-019-3316-z

**Published:** 2019-01-21

**Authors:** Jeremy V. Camp, Jolanta Kolodziejek, Norbert Nowotny

**Affiliations:** 1 Viral Zoonoses, Emerging and Vector-borne Infections Group, Institute of Virology, University of Veterinary Medicine Vienna, Vienna, Austria; 2 Department of Basic Medical Sciences, College of Medicine, Mohammed Bin Rashid University of Medicine and Health Sciences, Dubai, United Arab Emirates

**Keywords:** *Aedes japonicus japonicus*, *Culex pipiens*, Invasive mosquito, Vector competence, Flavivirus

## Abstract

**Background:**

The emergence of Usutu virus (USUV) in Europe was first reported in Austria, 2001, and the virus has since spread to many European countries. Initial outbreaks are marked by a mass die-off of European blackbirds (*Turdus merula*) and other bird species. During outbreaks, the virus has been detected in pools of *Culex pipiens* mosquitoes, and these mosquitoes are probably the most important enzootic vectors. Beginning in 2017, a second wave of blackbird deaths associated with USUV was observed in eastern Austria; the affected areas expanded to the Austrian federal states of Styria in the south and to Upper Austria in the west in 2018. We sampled the potential vector population at selected sites of bird deaths in 2018 in order to identify infected mosquitoes.

**Results:**

We detected USUV RNA in 16 out of 19 pools of *Cx. pipiens*/*Cx. torrentium* mosquitoes at sites of USUV-linked blackbird mortality in Linz and Graz, Austria. A disseminated virus infection was detected in individuals from selected pools, suggesting that *Cx. pipiens* form *pipiens* was the principal vector. In addition to a high rate of infected *Cx. pipiens* collected from Graz, a disseminated virus infection was detected in a pool of *Aedes japonicus japonicus*.

**Conclusions:**

We show herein that naturally-infected mosquitoes at foci of USUV activity are primarily *Cx. pipiens* form *pipiens*. In addition, we report the first natural infection of *Ae. j. japonicus* with USUV, suggesting that it may be involved in the epizootic transmission of USUV in Europe. *Ae. j. japonicus* is an invasive mosquito whose range is expanding in Europe.

## Background

Usutu virus (USUV) is a flavivirus (*Flaviviridae*) in the Japanese encephalitis virus serogroup originating from Africa [1]. In 2001, USUV was first identified in Austria, associated with a large die-off of Eurasian (or common) blackbirds (*Turdus merula* Linnaeus, 1758) [2], although the initial emergence in Europe may have been earlier [3]. Following the initial introduction, the virus spread to many European countries and is typically associated with the death of certain species of native birds, mainly blackbirds [4–7]. An observed reduction of bird deaths over time may be attributed to protection by herd immunity [8]. Despite this, there exists evidence of continued low-level virus activity in the years following the initial outbreaks in the form of bird seroconversion and the detection of viral nucleic acid in pools of mosquitoes [9, 10]. In 2016, USUV was reported from live and dead birds in Austria, Belgium, Germany, Hungary, France, Germany and the Netherlands [11, 12], as well as from human blood donors in Germany in 2017 [13] and in Austria from 2016–2018 [14, 15]. Therefore, USUV has established transmission in Europe.

The identification of USUV nucleic acid in field-captured mosquito pools suggests that *Culex pipiens* Linnaeus, 1758 is the principal vector in Europe [16]. In regions where West Nile virus (WNV, *Flaviviridae*) is endemic, USUV and WNV have been observed to co-circulate in an avian-mosquito transmission cycle [10, 16, 17]. Experimental vector competence studies have demonstrated that European *Cx. pipiens* form *pipiens* populations are competent vectors of USUV [18, 19]. However, it is unconfirmed if natural populations of *Cx. pipiens* are infected with USUV as only pooled adult females were tested in mosquito surveillance efforts and their infection status could not be determined [10, 16, 17].

Beginning in 2016, the presence of viral RNA in blood from human donors and in tissue samples from dead birds signaled increased transmission of USUV in Austria [12, 14]. In 2018, bird deaths in Austria increased over the prior year, and multiple USUV-infected blackbirds were confirmed from several sites including Linz, Upper Austria and Graz, Styria. Furthermore, obligatory seasonal blood donation screening in eastern Austria revealed 18 USUV infections among donors in 2018 [15], which is the highest number of human infections reported since the emergence of USUV in Austria in 2001 [2]. Recently, we reported the analysis of integrated human-vector-host surveillance for arboviruses in Austria [20]. Using this model, we performed targeted entomological investigations at sites where cases of blackbird deaths were confirmed to be linked to USUV infection. The goal was to determine the infection status of mosquitoes at sites of virus activity.

## Results

In total, 380 mosquitoes were collected from the two sites (Table 1). In Linz, 37 *Cx. pipiens*/*Cx. torrentium* Martini, 1925 were captured, 18 of which were gravid, and seven *Aedes japonicus japonicus* (Theobald, 1901) were collected (Table 1). In Graz, two nights of trapping resulted in 315 *Cx. pipiens*/*Cx. torrentium* (8 from the light trap, 2 of which were gravid, and all except for 32 of the remaining specimens collected in the gravid trap were gravid), 17 *Ae. j. japonicus* (10 from the light trap, and 2 of 7 from the gravid trap were gravid), three *Aedes vexans* (Meigen, 1830) captured in the light trap, and one *An. maculipennis* (Meigen, 1818) captured in the light trap (Table 1). Mosquitoes were pooled by site and species, and then tested for the presence of viral nucleic acids.

**Table 1:** Adult female mosquitoes collected from sites of Usutu virus-positive Eurasian blackbird deaths in Austria, 2018. Mosquitoes were collected overnight (Linz, one trap-night; Graz, two trap-nights) with a CDC miniature light trap baited with CO_2_ (LT) or a gravid trap containing hay infusion (GT)

Location	Species	Total	LT (gravid^a^)	GT (gravid^a^)	USUV+ /pools tested
Linz	*Aedes j. japonicus*	7	5 (0)	2 (0)	0/1
	*Culex pipiens*/*Cx. torrentium*	37	7 (3)	30 (15)	2/3^b^
Graz	*Ae. j. japonicus*	17	10 (0)	7 (2)	1/3^c^
	*Ae. vexans*	3	3 (0)	0 (0)	0/1
	*Anopheles maculipennis*	1	1 (0)	0 (0)	0/1
	*Cx. pipiens*/*Cx. torrentium*	315	8 (2)	307 (275)	14/16^d^
Total		380	34 (5)	346 (292)	17/25

^a^The number of gravid individuals from each trap is listed in parentheses following the total number

^b^Usutu virus nucleic acid was detected in two pools of *Cx. pipiens*/*Cx. torrentium* with 7 and 15 individuals, respectively

^c^Usutu virus nucleic acid was detected in one pool of six *Ae. j. japonicus* which was determined to be a disseminated infection

^d^Usutu virus nucleic acid was detected in 14 of 16 pools of *Cx. pipiens*/*Cx. torrentium* each containing between 7 and 25 individuals

Two of the three pools containing seven and 15 *Cx. pipiens*/*Cx. torrentium* mosquitoes, respectively, from Linz were positive for USUV nucleic acid (Table 1). Further testing of the individuals’ legs and wings revealed that the pool consisted of 2 *Cx. torrentium* and 5 *Cx. pipiens* form *pipiens*; USUV nucleic acid was found in the legs and wings of a single *Cx. pipiens* form *pipiens* individual (Table 2). Similarly, pooled bodies and pooled legs and wings from the 7 *Ae. j. japonicus* specimens captured in Linz were negative for flavivirus nucleic acid (Table 1).

**Table 2:** Molecular identification of *Culex* species with disseminated Usutu virus infection at foci of transmission in Austria, 2018

Species	USUV+ / tested	Linz	Graz
*Cx. pipiens* form *pipiens*	3/35	1/5	2/30
*Cx. torrentium*	0/2	0/2	0/0

*Notes*: The legs and wings from mosquitoes taken from Usutu virus-positive pools (USUV+) from each trap site (Linz: 1 pool with 7 mosquitoes; Graz: 2 pools with 15 mosquitoes each) were analysed individually to determine species and to detect if the infection was disseminated. The values are the number of individuals of a given species with a disseminated USUV infection / total individuals tested by trap site

From Graz, 14 of the 16 pools of *Cx. pipiens*/*Cx. torrentium* were positive for USUV nucleic acid (Table 1), all of which contained gravid individuals except for two pools consisting of 25 and 7 non-gravid individuals, respectively. The legs and wings from mosquitoes comprising two USUV-positive pools of 15 gravid *Cx. pipiens*/*Cx. torrentium* each were then tested individually. The pools consisted entirely of *Cx. pipiens* form *pipiens*, and USUV was detected in the legs and wings of two of the 30 *Cx. pipiens* form *pipiens*, indicating a disseminated infection (Table 2). In addition, USUV nucleic acid was detected in a pool of six *Ae. j. japonicus*; the legs and wings were tested separately and were positive for USUV nucleic acid, suggesting that the infection was disseminated.

Partial sequences within the NS5 gene of six USUV positive mosquito pools were determined, including 2 *Cx. pipiens*/*Cx. torrentium* pools from Linz (accession nos. MK121948 and MK121949), 3 *Cx. pipiens*/*Cx. torrentium* pools from Graz (accession nos. MK121944, MK121946 and MK121947) and 1 *Ae. j. japonicus* pool from Graz (accession no. MK121945). The sequences were 99.5–100.0% identical to each other and to the USUV sequences obtained from the birds found dead in the corresponding sites, all belonging to USUV cluster “Europe 2”. The sequence identities to the previous Austrian strains were between 99.2–100.0%. All mosquito pools tested negative for WNV.

## Discussion

In vector surveys, USUV is most frequently detected in pools of *Cx. pipiens*/*Cx. torrentium* [16]*.* However, in Italy for example, USUV nucleic acid was also identified in pools of the invasive mosquito, *Aedes albopictus* (Skuse, 1894), at relatively high frequency [21]. Other species of mosquitoes have been occasionally identified to be USUV-positive at a much lower frequency: *Anopheles maculipennis* (*s.l.*), *Culiseta annulata* (Schrank, 1776), *Ochlerotatus caspius* (Pallas, 1771) and *Ochlerotatus detritus* (Haliday, 1833) in Italy, and *Culex perexiguus* (Theobald, 1903) in Spain [16]. However, it is unknown whether these species are competent vectors. The ability to identify naturally infected vectors represents a challenge to the study of the enzootic transmission cycles of arboviruses. Additionally, female *Cx. pipiens* cannot be separated from *Cx. torrentium* by morphology, and therefore the detection of arboviral nucleic acid in mixed pools of *Cx. pipiens*/*Cx. torrentium* is ambiguous.

To address these challenges, we used bird deaths to identify foci of USUV transmission during the most recent outbreak in Austria. We used gravid traps to increase the likelihood that we would sample infected mosquitoes, i.e. those that have already fed upon viremic hosts. We tested for disseminated infection in selected individual mosquitoes by analysing legs and wings separately. This also allowed us to determine the species of mosquitoes that were infected with the virus, particularly to distinguish *Culex* spp. using molecular tests. We found disseminated infections in *Cx. pipiens* form *pipiens*, which others have determined is a competent vector species of USUV [18, 19], and thus this is most likely the principal vector involved in USUV transmission. Neither of the *Cx. torrentium* (*n* = 2) individuals were positive for USUV, although the number tested was much lower than the number of individual *Cx. pipiens* form *pipiens* tested (*n* = 35). The lower relative abundance of *Cx. torrentium* at the sites of virus activity here (Table 1) may suggest that they are not as important as *Cx. pipiens* form *pipiens* in enzootic transmission and maintenance of the virus.

In addition, we report the first natural infection of *Ae. j. japonicus* with USUV. In Austria, *Ae. j. japonicus* was first noted in southern Styria in 2011 near the Slovenian border and has also been reported from multiple countries in central Europe, including Switzerland and Italy [22–25]. It appears that multiple introductions into Europe have occurred [26] and the population in central Europe is aggressively expanding in range and local abundance [27]. It is a highly invasive mosquito and may displace endemic species where it is introduced [28]. Experimental studies have shown that *Ae. j. japonicus* is a competent vector of both WNV-lineage 1 in the USA [29, 30] and WNV-lineage 2 in Europe [31, 32], as well as chikungunya virus and dengue virus [33]. To our knowledge, the vector competence of *Ae. j. japonicus* for USUV has not yet been established.

Despite its wide distribution and high vector competence for many arboviruses, there is only a single report of a field population of *Ae. j. japonicus* being positive for WNV, identified in the USA during the initial outbreak of WNV [34]. *Ae. j. japonicus* has a strong preference for mammalian hosts [35–37], taking blood from many mammal species including humans [38]. Although avian blood meals have not been identified from field specimens, laboratory colonies take blood when offered captive birds [39]. Therefore it is unlikely that *Ae. j. japonicus* will be an important vector of enzootic transmission of USUV; however, this invasive species may be a bridge vector of USUV and/or WNV.

## Conclusions

Targeted entomological surveillance at foci of USUV-associated bird deaths supports the hypothesis that *Cx. pipiens* form *pipiens* is the major vector of USUV in Austria. The surveillance also identified that *Ae. j. japonicus*, an invasive species, was naturally infected with USUV.

## Methods

Through coordinated surveillance efforts, bird deaths in 2018 were investigated at the University of Veterinary Medicine Vienna [40]. Sites with four or more dead blackbirds testing positive for USUV were selected for targeted entomological surveillance. This included a site in Linz (Upper Austria; 48°17.001'N, 14°16.663'E; 1 trap-night) and a site in Graz (Styria; 47°04.995'N, 15°27.865'E; 2 trap-nights). Traps were set between one and three weeks following confirmed USUV-linked bird deaths. To sample the general mosquito population a CDC standard miniature light trap (“light trap”) baited with 1 kg of dry ice was used. In order to target the recently-infected mosquito population, an updraft gravid trap using a 10-day-old hay infusion as an oviposition attractant was used (both traps from J.W. Hock Co., Gainesville, FL, USA). Gravid traps baited with grass infusion are known to be effective sampling methods for both *Cx. pipiens* and *Ae. j. japonicus* [41]. Traps were set 1 h before sunset and collected 1 h after sunrise.

Trap contents were cooled for 2 min at -20 °C, and mosquitoes were sorted to species on dry ice using morphological identification keys [42, 43]. Mosquitoes were pooled by species, site, Sella stage, and trap-night. Species identifications were confirmed by molecular barcoding: a 684 bp portion of the mitochondrial cytochrome *c* oxidase 1 (*cox*1) gene was amplified by PCR (Go*Taq*® G2 PCR master mix, Promega, Mannheim, Germany) using VF1d and VR1d primers [44], sequenced by the Sanger method and compared to available sequences in GenBank. The legs and wings were removed from some specimens, selected haphazardly, and stored separately to test for a disseminated viral infection. Selected individual specimens identified as *Cx. pipiens*/*Cx. torrentium* were identified to species based on amplicon length polymorphism of the *Ace2* gene using primers ACEpip, ACEtorr and B1246s according to a published protocol [45]. To differentiate biotypes of *Cx. pipiens*, a 650 bp portion of the *cox*1 gene was amplified by PCR (primers COIF and COIR) and then digested with *Hae*III restriction enzyme (New England Biolabs, Frankfurt, Germany) according to a published protocol, which reveals a restriction site present in *Cx. pipiens* form *pipiens* but not in form *molestus* [46].

Mosquito pools or mosquito parts were homogenised in buffer on a bead mill (TissueLyser, Qiagen, Hilden, Germany), and nucleic acid was extracted from the cleared homogenate using a commercial kit (QIAamp viral RNA kit, Qiagen). Virus nucleic acid was amplified using real-time RT-PCR with a published ‘universal’ flavivirus primer set (PF1S and PF2Rbis) and SYBR green [47] (Luna®, New England Biolabs). Two virus-specific primer-probe sets were used to identify USUV or WNV nucleic acid [3, 48]. USUV-positive samples were further tested with conventional RT-PCR [4]. Amplicons were sequenced by Sanger sequencing (Microsynth Austria GmbH, Vienna, Austria), identified by nBlast search (https://blast.ncbi.nlm.nih.gov/Blast.cgi), and aligned with published USUV sequences from Austria (GenBank accession nos. MF063042, MF991886 and AY453411) in MEGA v.6 to determine sequence similarity.

## Abbreviations

USUV: Usutu virus
WNV: West Nile virus
